# Microaerobic insights into production of polyhydroxyalkanoates containing 3-hydroxyhexanoate via native reverse *β*-oxidation from glucose in *Ralstonia eutropha* H16

**DOI:** 10.1186/s12934-024-02294-4

**Published:** 2024-01-14

**Authors:** Kai-Hee Huong, Izumi Orita, Toshiaki Fukui

**Affiliations:** School of Life Science and Technology, Tokyo Institute of Technology, 4259 Nagatsuta, Midori-ku, Yokohama, 226-8501 Japan

**Keywords:** Microaerobic cultivation, Metabolic engineering, poly(3-hydroxybutyrate-*co*-3-hydroxyhexanoate), Polyhydroxyalkanoates, *Ralstonia eutropha* H16, Reverse *β*-oxidation

## Abstract

**Background:**

*Ralstonia eutropha* H16, a facultative chemolitoautotroph, is an important workhorse for bioindustrial production of useful compounds such as polyhydroxyalkanoates (PHAs). Despite the extensive studies to date, some of its physiological properties remain not fully understood.

**Results:**

This study demonstrated that the knallgas bacterium exhibited altered PHA production behaviors under slow-shaking condition, as compared to its usual aerobic condition. One of them was a notable increase in PHA accumulation, ranging from 3.0 to 4.5-fold in the mutants lacking of at least two NADPH-acetoacetyl-CoA reductases (PhaB1, PhaB3 and/or phaB2) when compared to their respective aerobic counterpart, suggesting the probable existence of (*R*)-3HB-CoA-providing route(s) independent on PhaBs. Interestingly, PHA production was still considerably high even with an excess nitrogen source under this regime. The present study further uncovered the conditional activation of native reverse *β*-oxidation (rBOX) allowing formation of (*R*)-3HHx-CoA, a crucial precursor for poly(3-hydroxybutyrate-*co*-3-hydroxyhexanoate) [P(3HB-*co*-3HHx)], solely from glucose. This native rBOX led to the natural incorporation of 3.9 mol% 3HHx in a triple *phaB*-deleted mutant (∆*phaB1*∆*phaB1*∆*phaB2-C2*)*.* Gene deletion experiments elucidated that the native rBOX was mediated by previously characterized (*S*)-3HB-CoA dehydrogenases (PaaH1/Had), β-ketothiolase (BktB), (*R*)-2-enoyl-CoA hydratase (PhaJ4a), and unknown crotonase(s) and reductase(s) for crotonyl-CoA to butyryl-CoA conversion prior to elongation. The introduction of heterologous enzymes, crotonyl-CoA carboxylase/reductase (Ccr) and ethylmalonyl-CoA decarboxylase (Emd) along with (*R*)-2-enoyl-CoA hydratase (PhaJ) aided the native rBOX, resulting in remarkably high 3HHx composition (up to 37.9 mol%) in the polyester chains under the low-aerated condition.

**Conclusion:**

These findings shed new light on the robust characteristics of *Ralstonia eutropha* H16 and have the potential for the development of new strategies for practical P(3HB-*co*-3HHx) copolyesters production from sugars under low-aerated conditions.

**Graphical Abstract:**

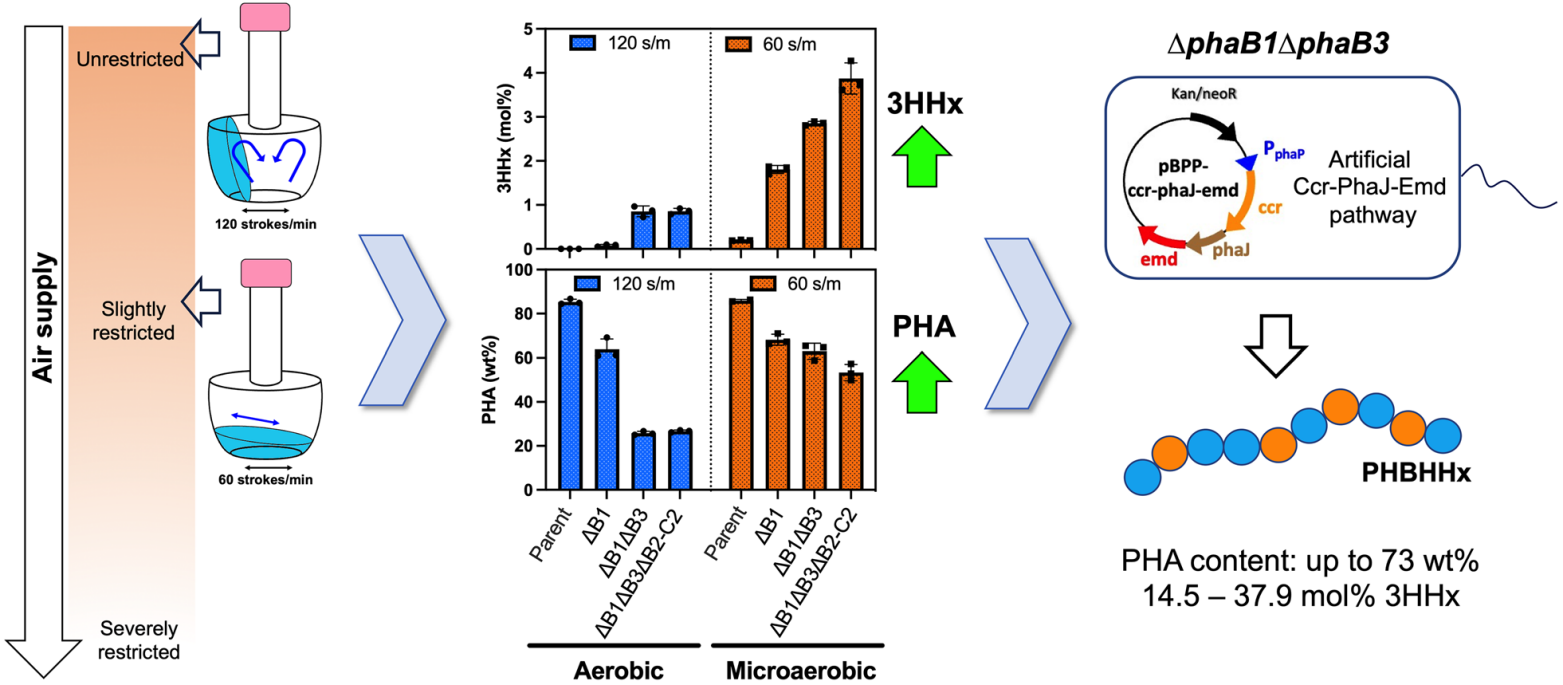

**Supplementary Information:**

The online version contains supplementary material available at 10.1186/s12934-024-02294-4.

## Background

Bacterial polyhydroxyalkanoates (PHAs) are bio-based polymeric materials that show high biodegradability not only in soil but also in the marine environments, and produced from renewable resources; making them promising materials that contribute to achieving Sustainable Development Goals (SDGs).

Poly(3-hydroxybutyrate-*co*-3-hydroxyhexanoate) [P(3HB-*co*-3HHx)] is a practical and by far the most implementable PHA that can be fabricated into various commercial products owing to its resemblance to conventional plastics such as low-density polyethylene and polypropylene [1]. To date, the copolymer has been industrially produced from plant oil under the trademark of Green Planet^™^ by KANEKA Co. Ltd., at the scale of over 5 thousand tons per year to support the growing demands in single-use plastics: cutlery, straw, container, coffee capsules, films, etc. [2]. In contrast to poly(3-hydroxybutyrate) [P(3HB)] homopolymer, P(3HB-*co*-3HHx) is characterized by reduced crystallinity and melting temperature, as well as improved flexibility attributed with the longer side-chain in the 3HHx (C_6_) comonomer [3].

The copolymer was initially discovered to be synthesized by *Aeromonas caviae* FA440 having the biosynthetic genes clustered as *phaP-C-J*_*Ac*_ encoding phasin, a unique class I PHA synthase accepting C_4_-C_7_ monomers, and (*R*)-specific enoyl-CoA hydratase, respectively, from plant oils and fatty acids as substrates [4–6]. Efficient production of this copolymer from plant oils has been achieved by engineering of high PHA-performing *Ralstonia eutropha* (*Cupriavidus necator*) [7, 8], which were the modification of *β*-oxidation and (*R*)-3HB-CoA-formation pathways as well as introduction of the double mutant (N149S/D171G) of PhaC_*Ac*_ (PhaC_NSDG_) [5, 9–12]. The catalytic properties of (*R*)-specific enoyl-CoA hydratase PhaJ, linking *β*-oxidation and PHA biosynthesis, is one of key factors regulating 3HHx composition in the resulting PHA polymers.

Metabolic engineering for biosynthesis of P(3HB-*co*-3HHx) from structurally unrelated sugars is another important technology for the cost-effective bioproduction; considering that sugars are relatively inexpensive and can be the alternative to the bioprocess involving plant oils that usually causes severe foaming and complicates the downstream processing [13]. The intracellular formation of (*R*)-3HHx-CoA from sugars has been achieved by artificial reverse *β*-oxidation (rBOX) pathway in which key enzymes are bacterial NADPH-dependent crotonyl-CoA carboxylase/reductase (Ccr) and mammalian ethylmalonyl-CoA decarboxylase (designated as Emd) [14–16]. The Ccr-Emd combination plays a role by connecting crotonyl-CoA formed from acetyl-CoA to butyryl-CoA that is then elongated and converted to (*R*)-3HHx-CoA. Namely, the bifunctional Ccr catalyzes reduction of crotonyl-CoA to butyryl-CoA as well as reductive carboxylation of crotonyl-CoA to ethylmalonyl-CoA, and Emd converts ethylmalonyl-CoA back into butyryl-CoA. The *R. eutropha* strains equipped with the artificial pathway effectively produced P(3HB-*co*-3HHx) from fructose or glucose [14, 15]. The recent study has demonstrated that the artificial pathway driven by Ccr-Emd is also functional chemolitoautotrophically in the engineered *R. eutropha*, enabling the gas fermentation of P(3HB-*co*-3HHx) using CO_2_ and H_2_ as carbon and energy sources, respectively [17]. The progress of P(3HB-*co*-3HHx) production from structurally unrelated carbon sources and the relevant enzymes are summarized in Additional file 1: Table S1.

We here discovered that *R. eutropha* possesses a native de novo biosynthesis pathway for (*R*)-3HHx-CoA and NADPH-acetoacetyl-CoA reductases (PhaBs)-independent pathway for provision of (*R*)-3HB-CoA, functional under microaerobic conditions. Despite the numerous studies on *R. eutropha* as a useful host for microbial cell factories, there have been limited information for bioproduction using this bacterium under low-aerobic or microaerobic conditions. These results reflect the metabolic versatility of *R. eutropha* as well as arises the high potential of this bacterium as a valuable platform from the industrial biomanufacturing point of view.

## Results

### Unusual P(3HB-*co*-3HHx) biosynthesis profile of *R. eutropha* under low-aerobic condition

**Fig. 1:**
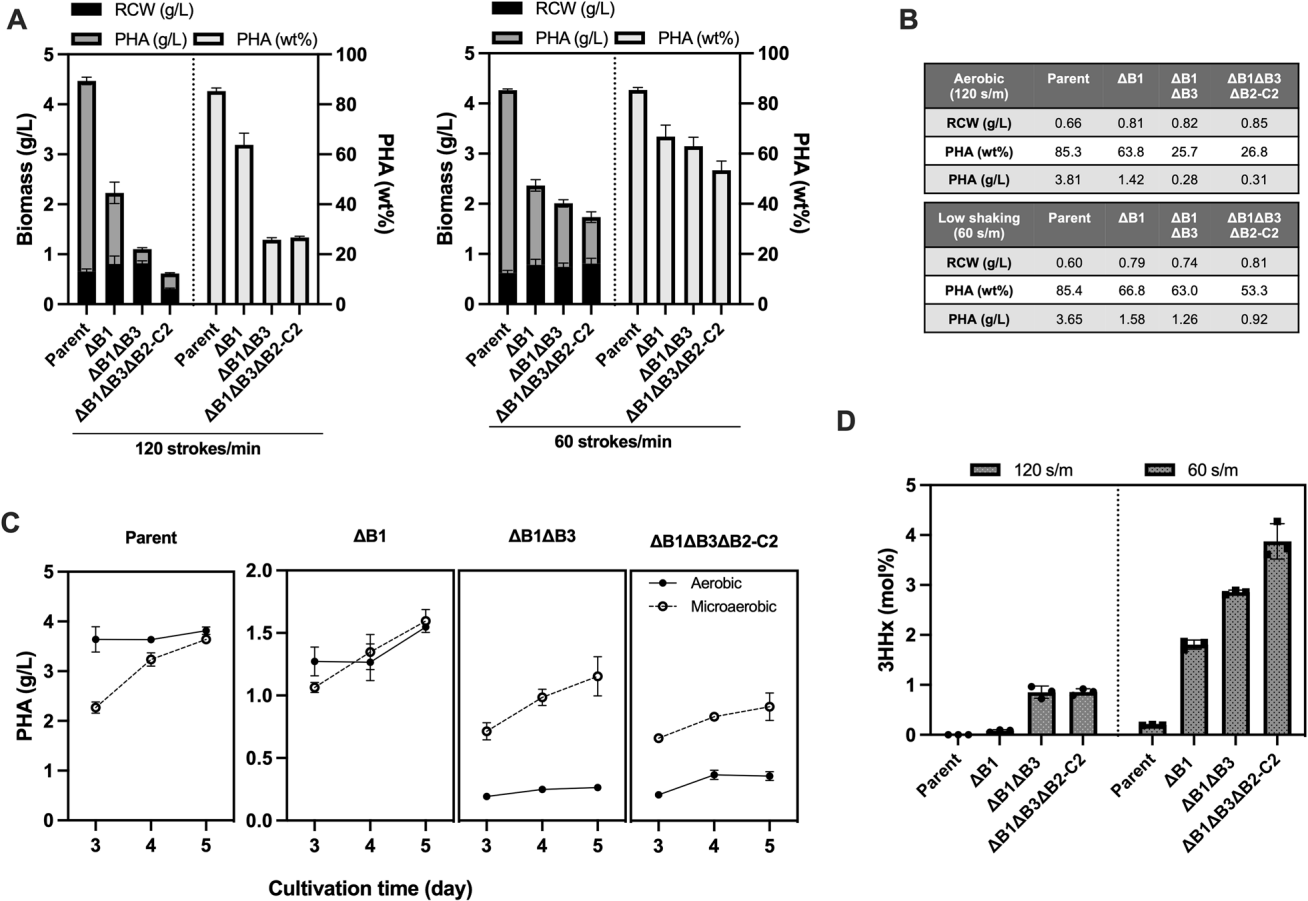
**A** Production of P(3HB-*co*-3HHx) from 1% (w/v) glucose by *R. eutropha* strains under aerobic (120 strokes/min) and low-shaking (60 strokes/min) conditions for 120 h. **B** The final cell growth [RCW (residual cell weight)] (g/L), PHA content (wt%) and PHA concentration (g/L) after the 120 h cultivation. **C** Time-course of PHA concentration during the PHA accumulation phase. **D** 3HHx compositions in the accumulated PHA under aerobic and low-aerobic conditions. The strains are NSDG-GG (parent), ΔB1 (∆*phaB1*), ΔB1ΔB3 (∆*phaB1*∆*phaB3*), and ΔB1ΔB3ΔB2-C2 (∆*phaB1*∆*phaB3*∆*phaB2-C2*). Data are represented as mean values ± standard deviation of three replicates

Among the three PhaB paralogs in *R. eutropha* H16 [18], it has been reported that the highly-expressed PhaB1 is a major reductase and the weakly expressed PhaB3 fairly compensated P(3HB) synthesis when PhaB1 was absent [19]. During our investigation for PHA copolymer biosynthesis by engineered strains of *R. eutropha*, we noticed that *phaB*-deleted strains showed altered PHA biosynthesis property when shaking rate was reduced to 60 strokes/min from usual 120 strokes/min (Fig. 1, Additional file 1: Table S4). The left panel in Fig. 1A shows PHA biosynthesis from glucose by the glucose-assimilating *R. eutropha* strain NSDG-GG harboring *phaC*_NSDG_ and its respective *phaB*-deleted variants, denoted as parent (NSDG-GG), ΔB1 (∆*phaB1*), ΔB1ΔB3 (∆*phaB1*∆*phaB3*) and ΔB1ΔB3ΔB2-C2 (∆*phaB1*∆*phaB1*∆*phaB2-C2*). The triple mutant ΔB1ΔB3ΔB2-C2 was constructed by deletion of *phaB2* along with *phaC2* encoding the second PHA synthase with unknown physiological function, since they are adjacent to each other. Under the aerobic condition (120 strokes/min), the cellular PHA content was reduced from 85 wt% to 64 wt% by deletion of *phaB1*, and further decreased to 26 wt% by double deletion of *phaB1* and *phaB3*. Additional deletion of *phaB2-phaC2* did not affect the PHA synthesis. These results were consistent with those reported by Budde et al. [19].

Interestingly, relatively higher amount of PHA (63 and 53 wt%) was produced under the slow-shaking condition (60 strokes/min) even by the double and triple *phaB*-deletants, respectively (right panel in Fig. 1A, B). The single deletion mutant ΔB1 showed slow PHA formation under this condition but outperformed the aerobic condition after 96 h; meanwhile the double and triple deletants could still produce significant amount of PHA under the low-aerobic cultivation (Fig. 1C, Additional file 1: Table S5), with 4.5-fold production (> 60 wt%) by the double *phaB*-deleted mutant and 3.0-fold (> 50 wt%) by the triple *phaB*-deletant when compared to the aerobic counterpart.

PHA produced by the ΔB1 strain was nearly P(3HB) homopolymer containing negligible fraction of 3HHx (< 0.1 mol%) under the usual aerobic condition as observed so far, and the additional deletion of *phaB3* led to trace but stagnant 3HHx composition (~ 1 mol%). We here found that, under the low aerated condition, the 3HHx fractions in PHA became significant (1.8 mol%) for the ΔB1 strain and they were further increased to 2.9 and 3.9 mol% by the double and triple deletion of *phaB* isologs, respectively (Fig. 1D). These results indicated the presence of native pathway for formation of (*R*)-3HHx-CoA from acetyl-CoA precursor in *R. eutropha*, which was independent from PhaB and activated during the low-aerobic cultivation.

### Revisiting PHA induction mode in *R. eutropha*: the effects of nitrogen and oxygen limitation

**Fig. 2:**
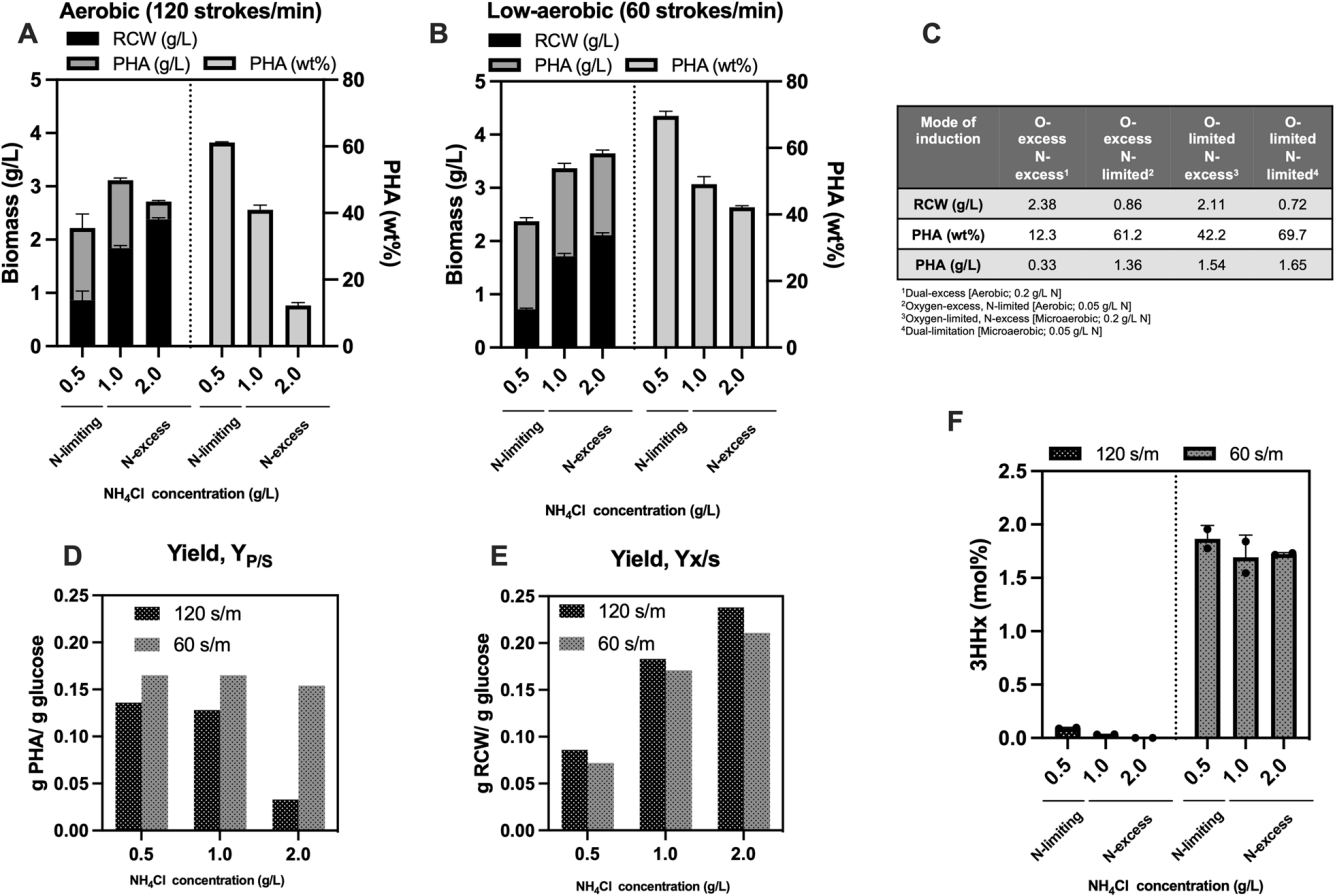
Effects of concentration of nitrogen source (0.5, 0.1 and 0.2 g/L) on PHA accumulation by *R. eutropha* ΔB1 strain under aerobic condition (oxygen-excess) (**A**) and low-aerobic condition (oxygen-limiting) (**B**). Supplementation of 0.5 g/L NH_4_Cl was considered nitrogen-limiting condition; and those of 1.0 and 2.0 g/L NH_4_Cl were defined as nitrogen-excess condition. **C** The final cell growth [RCW (residual cell weight)] (g/L), PHA content (wt%), and PHA concentration (g/L) after the 120 h cultivation. **D** PHA yield to glucose [Y_P/S _(g-PHA/g-glucose)]. **E** Cell yield to glucose [Y_X/S _(g-residual cell weight/g-glucose)]. **F** 3HHx compositions in the accumulated PHA under aerobic and low-shaking conditions. Data are represented as mean values ± standard deviation of two replicates

While PHA biosynthesis is usually induced under unbalanced growth lacking of nitrogen source [4, 9, 10, 12, 20], the present microaerobic PHA production by *R. eutropha* was done on dual nitrogen and oxygen limitations. We thus investigated the production behavior of the single *phaB1*-deleted ΔB1 strain, on aerobic (O-excess) and low-aerobic (O-limiting) at a varying concentration of nitrogen source (N-excess and N-limiting) (Fig. 2, Additional file 1: Table S6).

Under the aerobic condition, nitrogen limitation was necessary to induce PHA production and increased nitrogen source constituted to the balanced growth that resulted in reduction of PHA accumulation, as observed so far (Fig. 2A). In contrast, under the low-aerated condition, the increasing NH_4_Cl from 0.5 to 2.0 g/L marked a significant increase in bacterial growth (0.72–2.11 g/L) but only a slight decrease in PHA concentration (1.65–1.53 g/L) (Fig. 2B). This implied that PHA synthesis was still induced regardless of nitrogen concentration when oxygen was restricted. It is also worthwhile to mention that total biomass obtained during the low-shaking cultivation tended to be higher than that by the aerobic cultivation, notably under the nitrogen-excess condition (Fig. 2A–C). The PHA yield Y_P/S_ (g-PHA/g-glucose) and cell yield Y_X/S_ (g-residual cell/g-glucose) in Fig. 2D, E indicated higher magnitude for PHA production than aerobic one. The 3HHx compositions were remained to be significant (~ 1.6 mol%) throughout the variation of nitrogen amount under the low-aerobic condition (Fig. 2F).

Biosynthesis of PHA based on oxygen limitation has been rare for *R. eutropha* but was investigated under chemolithoautotrophic conditions using H_2_ and CO_2_ [21–23]. The present results in Fig. 2 coincided with those reported by Ishizaki and Tanaka [22] that an O-limiting–N-excess condition yielded high cell growth with moderate PHA content and consequent high PHA production, suggesting the shared PHA production mechanism in the different trophic modes.

### Identification of genes responsible for the native rBOX of (*R*)-3HHx-CoA de novo biosynthesis from glucose under low-aerobic conditions

**Fig. 3:**
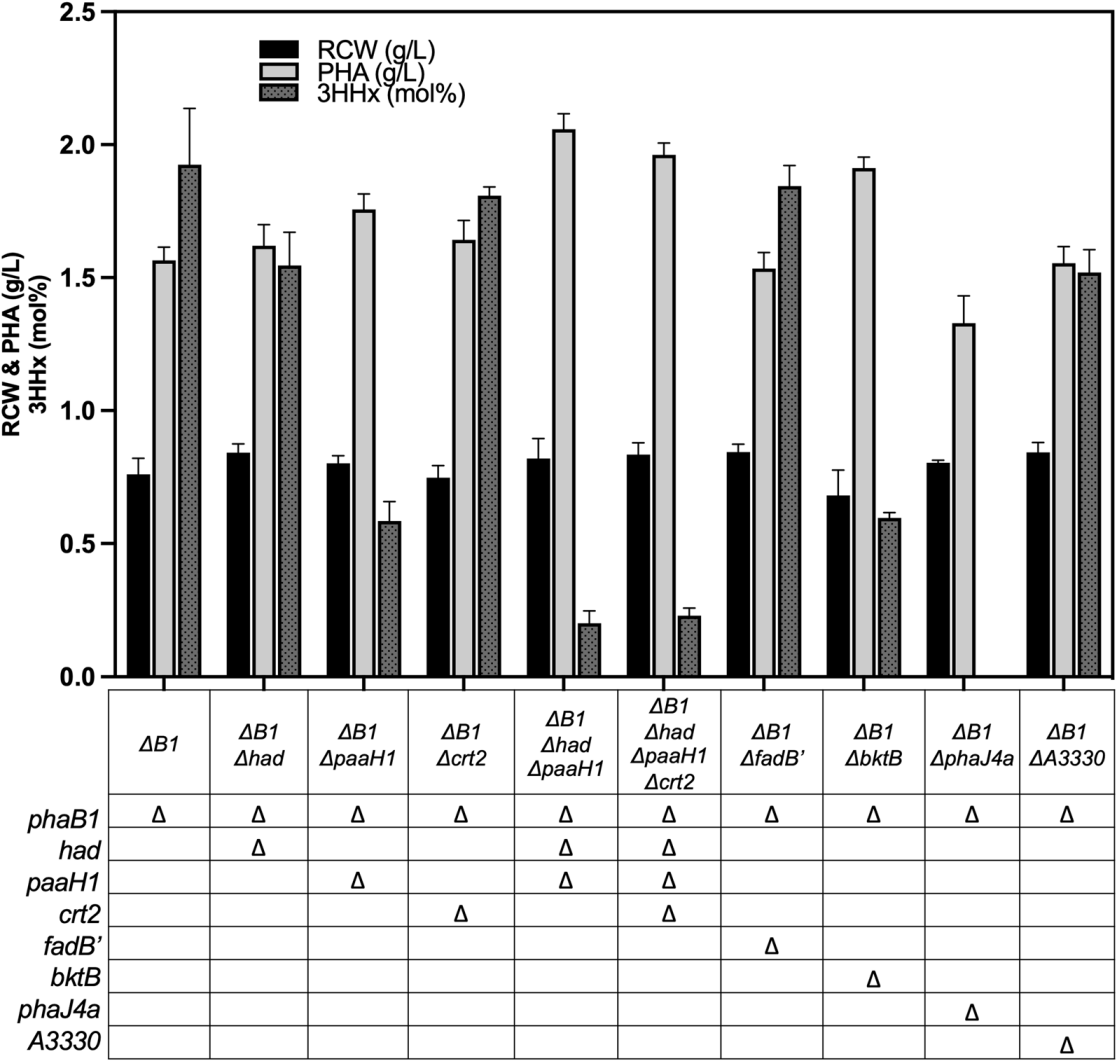
Effects of disruption of genes potentially related to (*R*)-3HHx-CoA formation in *R. eutropha* on P(3HB-*co*-3HHx) biosynthesis from glucose under the low-shaking condition*.* The gene names and the translated enzymes are summarized in Additional file 1: Table S2. Data are represented as mean values ± standard deviation of three replicates

A series of mutants were constructed based on the ∆B1 strain by disrupting endogenous genes potentially responsible to (*R*)-3HHx-CoA formation, and subjected to low-shaking cultivation (Fig. 3, Additional file 1: Table S7). The two (*S*)-3HB-CoA dehydrogenases PaaH1 (H16_A0282) and Had (H16_A0602), as well as the (*S*)-specific crotonase Crt2 (H16_A3307) in *R. eutropha* were reported to be broad substrate specific [20], thus possess capability to function in conversion of 3-oxoacyl-CoA to *trans*-2-enoyl-CoA via (*S*)-3-hydroxyacyl-CoA of C_4_ and C_6_. The gene deletion analyses indicated the crucial roles of the two dehydrogenases in the (*R*)-3HHx-CoA formation, as the C_6_ composition was decreased to 0.6 mol% by the single deletion of *paaH1* and to 0.2 mol% by the double deletion of *paaH1* and *had*. Neither change in cell growth nor PHA synthesis was observed by the deletion of Crt2, unexpectedly. Unlike under usual aerobic condition [15], the introduction of a tandem of *had-crt2* by using a broad host range-expression vector or by replacement of *phaB1* in the chromosomal *pha* operon did not affect the 3HHx composition in the resulting PHA (data not shown). Considering the similar catalytic properties of Had and PaaH1 to each other, the dehydrogenation of 3-oxohexanoyl-CoA was thought to be not the rate-limiting step in the (*R*)-3HHx-CoA formation under the low-aerobic condition. FadB’ (H16_A0461) which is bifunctional (*S*)-3-hydroxyacyl-CoA dehydrogenase/(*S*)-crotonase in *β*-oxidation [14] also showed no changes as well upon the gene deletion.

BktB is one homolog of *β*-ketothiolases with broad substrate specificity and has been reported to be important for condensation of acetyl-CoA and propionyl-CoA/*n*-butyryl-CoA to form 3-oxoacyl-CoAs of C_5_-C_6_ in the biosynthesis of PHA copolymers [6, 24]. Under the microaerobic condition, the deletion of *bktB* markedly decreased the 3HHx incorporation (0.6 mol%), implying partial significance of *bktB* in the pathway as well as function of other thiolase paralog(s) for the condensation. Kawashima et al. [25] demonstrated that PhaJ4a (H16_A1070) was the major (*R*)-enoyl-CoA hydratase in *R. eutropha* that supplies (*R*)-3HHx-CoA through aerobic *β*-oxidation on soybean oil. Here, the disruption of *phaJ4a* resulted in complete block of (*R*)-3HHx-CoA formation in the glucose-fed low-aerobic condition.

The ability of *R. eutropha* to supply (*R*)-3HHx-CoA from glucose under the microaerobic condition indicated the presence of unknown enzyme(s) catalyzing conversion of crotonyl-CoA to butyryl-CoA prior to the chain elongation. In the KEGG database, H16_A3330 is annotated as acryloyl-CoA reductase (NADPH), possibly catalyzing the reduction of the double bond in short-chain 2-enoyl-CoAs. Nevertheless, the *h16_A3330*-deleted strain ΔB1ΔA3330 showed a slight decrease in 3HHx fraction to 1.5 mol%, suggesting only partial participation of this reductase in the formation of butyryl-CoA.

### Concerted effect of the exogenous Ccr-PhaJ-Emd with native rBOX on P(3HB-co-3HHx) synthesis under low-aerobic cultivation

**Fig. 4:**
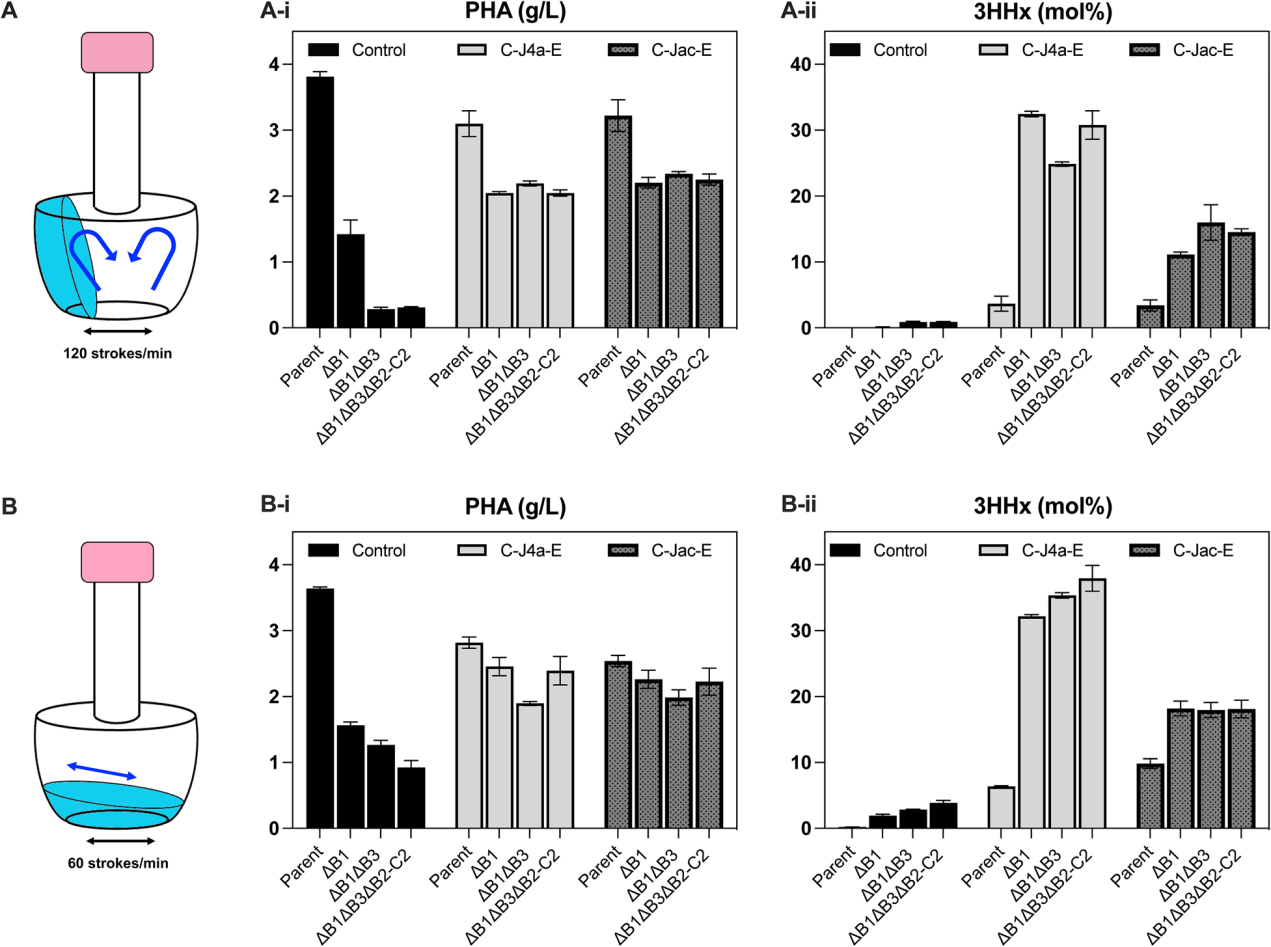
Effect of introduction of Ccr-Emd combination along with PhaJ on P(3HB-*co*-3HHx) biosynthesis by *R. eutropha* strains from glucose under (**A**) aerobic (120 s/m) and (**B**) low-aerobic (60 s/m) conditions*.* The strains are NSDG-GG (parent), ΔB1 (∆*phaB1*), ΔB1ΔB3 (∆*phaB1*∆*phaB3*), and ΔB1ΔB3ΔB2-C2 (∆*phaB1*∆*phaB3*∆*phaB2-C2*). CJ4aE*,* pBPP-ccr_Me_-phaJ4a-emd; CJ_Ac_E*,* pBPP-ccr_Me_-phaJ_Ac_-emd*.* Data are represented as mean values of three replicates

The effects of the artificial rBOX, driven by Ccr_*Me*_, PhaJ and Emd_*Mm*_ on P(3HB-*co*-3HHx) biosynthesis by *R. eutropha* under the low-aerobic condition were then investigated (Fig. 4, Additional file 1: Table S8). The expression plasmids pBPP-ccr_Me_-phaJ4a-emd and pBPP-ccr_Me_-phaJ_Ac_-emd were used for this purpose, in which *phaJ4a*_*Re*_ and *phaJ*_*Ac*_ are the genes of (*R*)-specific enoyl-CoA hydratase specific to medium-chain-length and short-chain-length substrates, respectively. In addition to the significant increase in the 3HHx composition up to 6.4 ~ 9.8 mol% in the parental NSDG-GG by the vectors, the compositional change was more compelling in all the *phaB*-deleted mutants, as shown by the increase in 3HHx composition up to 32 ~ 38 mol% and 18 mol% by introduction of the vectors harboring *phaJ4a* and *phaJ*_*Ac*_, respectively. The results demonstrated the concerted action of the artificial and the native rBOX for formation of (*R*)-3HHx-CoA under the low-aerobic condition. The high 3HHx composition in the resulting copolyester by co-expression of PhaJ4a was agreed with the preference of PhaJ4a towards medium-chain-length 2-enoyl-CoA substrates.

**Fig. 5:**
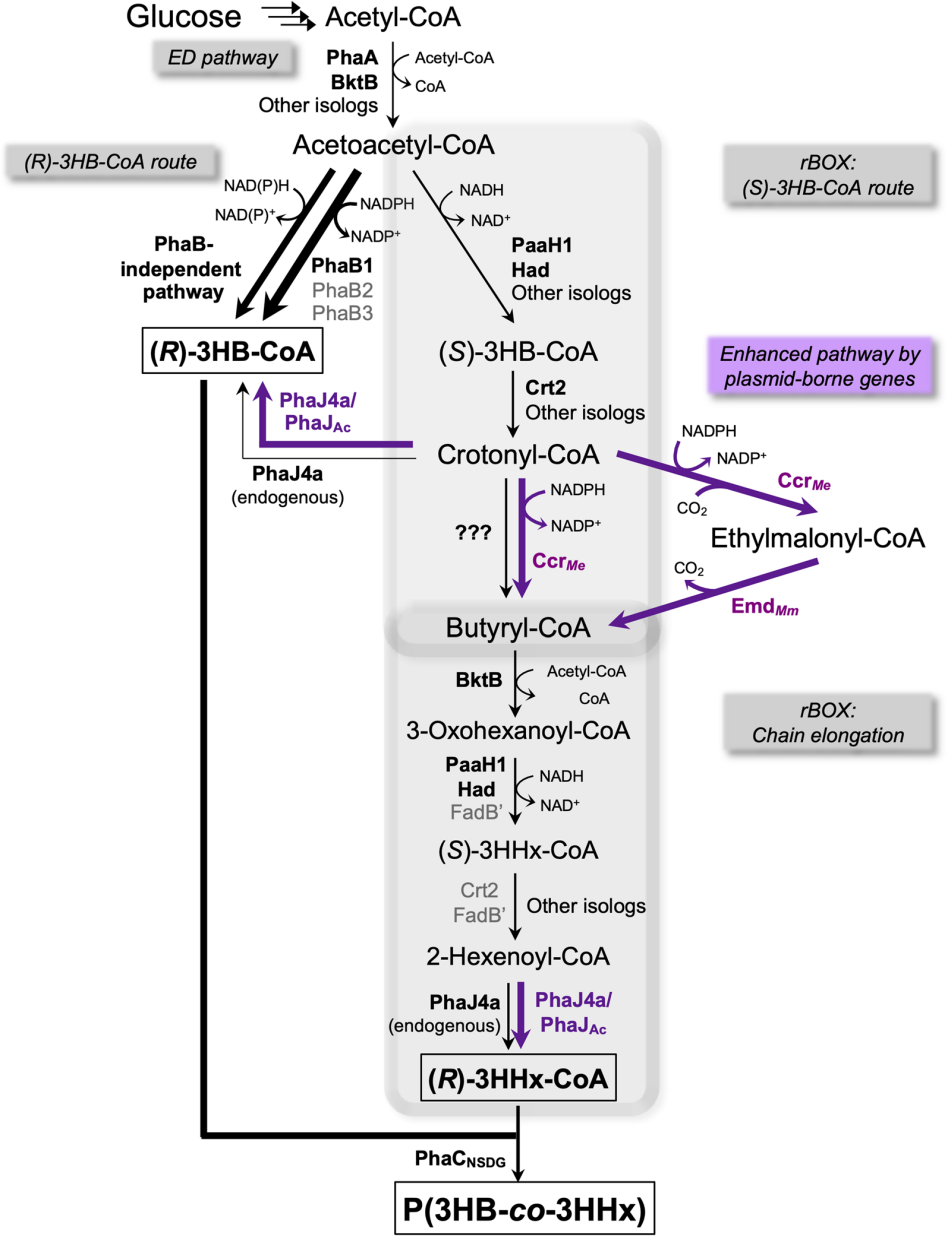
Metabolic pathways for P(3HB-*co*-3HHx) biosynthesis in *R. eutropha* under the low-aerated conditions. Promoted PHA biosynthesis in *R. eutropha* H16 under slow-shaking condition could be due to the presence of PhaB-independent pathway such as by enigmatic NAD(P)H-dependent (*R*)-specific reductase, or (*S*)-specific route mediated by other isologs of (*S*)-3HB-CoA dehydrogenase(s) and crotonase(s). The microaerobic cultivation has conditionally activated the native rBOX pathway as well, leading to the formation of (*R*)-3HHx-CoA. Further introduction of heterologous Ccr-PhaJ-Emd (artificial rBOX) showed concerted effect with native rBOX on P(3HB-*co*-3HHx) production under aforementioned condition. Black arrows indicate the native pathways whereas purple arrows indicate the plasmid borne artificial pathway

The introduction of the either expression vector collectively restored the PHA production capability in all the *phaB*-deletants under both aerobic and microaerobic cultivation. This is most probably due to PhaJ-catalyzed conversion of crotonyl-CoA to (*R*)-3HB-CoA in addition to the conversion of 2-hexenoyl-CoA to (*R*)-3HHx-CoA (Fig. 5). The improvement was drastic in double (ΔB1ΔB3) and triple *phaB*-deleted (ΔB1ΔB3ΔB2-C2) strains, with the overall PHA concentration comparable to ΔB1 strain (1.9–2.4 g/L).

## Discussion

This study demonstrated that a low-aerobic or microaerobic condition with slow-shaking of the media promoted PHA biosynthesis in *R. eutropha* regardless nitrogen limitation, and moreover led to conditional activation of native reverse *β*-oxidation (rBOX) pathway. This condition was still able to support the bacterial growth as shown in the similar residual cell weight between both shaking conditions. It has been known that P(3HB) functions as an electron sink to maintain cellular redox balance under anaerobic conditions in several facultative anaerobes [26]. Usually, when oxygen availability is restricted, the cells need other pathway(s) to regenerate oxidative cofactors from the reductive form, thus NADPH-dependent reduction of acetoacetyl-CoA to (*R*)-3HB-CoA plays the role in the cofactor regeneration in P(3HB)-producing anaerobes. In the present case of *R. eutropha*, the oxygen respiration was not fully retarded because the cell yield (Y_x/s_) under the low-shaking conditions was only slightly lower when compared with those under the usual aerobic conditions (Fig. 2E). The excess reducing equivalents not regenerated by the respiration under the limited oxygen availability would promote PHA biosynthesis to balance the cellular redox state. In fact, NADH was accumulated in *R. eutropha* when the terminal electron acceptor O_2_ was limited under the anoxic condition [27]. The improved PHA biosynthesis under the oxygen limitation has also been seen for *Azotobacter beijerinckii* [28], *Azotobacter vinelandii* [29], *Allochromatium vinosum* [30] and halophilic bacterium *Halomonas bluephagenesis* [31].

Our study also demonstrated a striking difference in PHA accumulation trend between aerobic and low-aerobic cultivation. PhaB1 is the major acetoacetyl-CoA reductase for (*R*)-3HB-CoA formation in both the conditions. PhaB3 is the minor reductase under usual aerobic conditions as reported previously [19], however, this is not applicable under the low-aerobic condition since the disruption of *phaB3* resulted in only slight reduction in PHA production. Given the fact that the double and triple *phaB*-deletants could still produce significant amount of PHA (Fig. 1), it was suggested that *R. eutropha* possesses other enzyme(s) for the formation of (*R*)-3HB-CoA from acetoacetyl-CoA functional under the low oxygen condition. rBOX for the C_4_-intermediates potentially participated in the PhaB-independent formation of (*R*)-3HB-CoA via (*S*)-3HB-CoA with the aid of (*R*)-2-enoyl-CoA hydratase(s). Nevertheless, PaaH1, Had, and Crt2 were not the major enzymes contributing to the (*R*)-3HB-CoA formation. PhaJ4a seemed to partially play the role, as the gene deletion of *phaJ4a* slightly reduced the PHA production in the low-shaking cultivation. Alternatively, unidentified (*R*)-specific reductase such as some isologs of FabG [NADPH 3-oxoacyl-acyl carrier protein (ACP) reductase] [32] and PhaG (3-hydroxyacyl-ACP thioesterase) along with CoA-ligase [32], or enigmatic NADH-dependent (*R*)-reductase may function in providing (*R*)-3HB-CoA from acetoacetyl-CoA in *R. eutropha* under such condition. Investigation such as comparative transcriptomics analysis and gene deletion studies might be required to identify the possible pathways contributes to the microaerobic-mediated (*R*)-3HB-CoA formation.

So far, *R. eutropha* has been believed to lack a pathway for formation of (*R*)-3HHx-CoA from sugar-derived acetyl-CoA molecules, because this bacterium produced only P(3HB) homopolymer from sugars even when a heterologous PHA synthase exhibiting broad substrate specificity (such as PhaC_NSDG_) was expressed within the cells. The present results indicated the rBOX for formation of (*R*)-3HHx-CoA from C_4_-acyl-CoA intermediates was functional specifically under the low-shaking condition. It was supposed that the robust *β*-oxidation in *R. eutropha* with multiple isologs enables the function of rBOX and the resulting native ability to form (*R*)-3HHx-CoA directing to the copolyester biosynthesis, when needed. The gene disruption analysis for several known enzymes demonstrated the actual functions of BktB, PaaH1/Had, and PhaJ4a in the rBOX pathway for the C_6_-intermediares under the low-aerated condition, whereas Crt2 and bifunctional FadB’ did not contribute to it. The conditional activation of rBOX would be also related to the reduced availability of oxygen. Namely, the reduction of crotonyl-CoA and 3-oxohexanoyl-CoA in rBOX would play the role in redox homeostasis, in addition to the reduction of acetoacetyl-CoA to (*R*)-3HB-CoA, as described above (Fig. 5). As shown in the previous artificial pathway for biosynthesis of P(3HB-*co*-3HHx) from structurally unrelated sugars [14, 15], the key reaction is the reduction of crotonyl-CoA to butyryl-CoA for thiolase-mediated elongation of C_4_ to C_6_. However, the native enzyme(s) responsible for the reduction of crotonyl-CoA in *R. eutropha* has been unclear. Although a putative acryloyl-CoA reductase (H16_A3330) had been one candidate for the unidentified reductase, the gene disruption denied the participation of H16_A3330 as a major enzyme in the rBOX pathway. Further investigation is required to identify the missing reductase in *R. eutropha*.

With the aid of artificial pathway driven by heterologous Ccr_*Me*_ and Emd_*Mm*_ along with PhaJ via plasmid expression, copolyesters with higher 3HHx monomer composition could be achieved under the low-aerated condition when compared to the corresponding aerobic cultivation (Fig. 4). The results suggested that the crotonyl-CoA reduction step mediated by the unknown native reductase was not sufficient, as well as indicated the importance of chain length-specificity of PhaJ in regulating the 3HHx composition in the resulting copolyesters. The similar trend has also been observed in a recent report focusing on autotrophic production of P(3HB-*co*-3HHx) by the engineered *R. eutropha*. Tanaka et al. [17] conducted the autotrophic cultivation of *R. eutropha* harboring pBPP-ccr_Me_-phaJ_4a_-emd_Mm_ on the gas mixture of H_2_/O_2_/CO_2_ (8:1:1), where the oxygen concentration was set to low both to induce PHA synthesis and avoid the risk of hydrogen explosion. They achieved efficient production of P(3HB-*co*-3HHx) from CO_2_ and H_2_ with higher 3HHx monomer compositions of 44–48 mol% than those obtained by the same strain cultivated on fructose. This was probably due to synergistic correlation between the native rBOX pathway activated under low-aerobic environment and the artificial rBOX, demonstrating one of examples for the usefulness of the application of PHA production under micro-aerobic condition.

## Conclusions

Usual bioprocesses using aerobic microbes highly demand oxygen with low solubility in the aqueous media to support efficient cell growth and bioconversion, thus high transfer coefficient of oxygen is achieved by vigorous aeration and/or agitation requiring much energy. This work demonstrated that the low-aerobic cultivation could promote the PHA biosynthesis in *R. eutropha* H16-derived strains, particularly in the *phaBs*-lacking mutants. Moreover, it was found that the low-aerobic condition enabled P(3HB-*co*-3HHx) biosynthesis mediated by the native rBOX, and exogenous Ccr-PhaJ-Emd (artificial rBOX) showed synergistic effect on the (*R*)-3HHx-CoA formation. The knowledge obtained by the current study is expected to be useful for compositional regulation of PHA copolyesters produced not only from sugars but CO_2_ as well, considering the natural property of *R. eutropha* as the knallgas bacterium.

## Materials and methods

### Bacterial strains and plasmids

The bacterial strains and plasmids used in this study are listed in Table 1. *R. eutropha* strains were cultivated at 30 °C in a nutrient-rich (NR) medium containing 10 g of bonito extract (Kyokuto, Tokyo, Japan), 10 g of polypeptone, and 2 g of yeast extract in 1 L of tap water. *E. coli* strains were grown at 37 °C in a Lysogeny broth (LB) medium for general gene manipulation and transconjugation. Kanamycin (100 mg/L) was added to the medium when necessary.

**Table 1:** Strains and plasmids used in this study

Strains/plasmids	Description/genotype	References
Strains		
*Escherichia coli*		
S17-1	*thi pro hsdR recA* chromosomal RP4; Tra^+^; Tmp^r^ Str/Spc^r^	Simon et al. [33]
*Ralstonia eutropha*		
H16	Wild type	DSM 428
NSDG-GG	H16 derivative; ∆*phaC*::*phaC*_NSDG_, *ΔnagR, nagE*(G793C),* P*_*A2858*_* -glpFK*_*Ec*_*-h16_A2858*	Zhang et al. [15]
ΔB1	NSDG-GG derivative; Δ*phaB1*_*Re*_	Mifune et al. [34]
ΔB1ΔB3	NSDG-GGΔB1 derivative; Δ*phaB3*	This study
ΔB1ΔB3ΔB2-C2	NSDG-GGΔB1ΔB3 derivative; Δ*phaB2-phaC2*	This study
ΔB1Δhad	NSDG-GGΔB1 derivative; Δ*had*	This study
ΔB1ΔpaaH1	NSDG-GGΔB1 derivative; Δ*paaH1*	This study
ΔB1Δcrt2	NSDG-GGΔB1 derivative; Δ*crt2*	This study
ΔB1ΔhadΔpaaH1	NSDG-GGΔB1Δhad derivative; Δ*paaH1*	This study
ΔB1ΔhadΔpaaH1Δcrt2	NSDG-GGΔB1ΔhadΔpaaH1 derivative; Δ*crt2*	This study
∆B1∆A3330	NSDG-GGΔB1 derivative; Δ*h16_A3330*	This study
ΔB1ΔbktB	NSDG-GGΔB1 derivative; Δ*bktB*	This study
ΔB1ΔphaJ4a	NSDG-GGΔB1 derivative; Δ*phaJ4a*	This study
∆B1∆fadB’	NSDG-GGΔB1 derivative; Δ*fadB’*	This study
Plasmids		
pK18mobsacB	pMB1 *ori*, *mob*, Kan^r^, *sacB*	Schafer et al. [35]
pk18msΔB3	pK18mobsacB derivative; *phaB3 del*	Insomphun et al. [14]
pk18msΔB2-C2	pK18mobsacB derivative; *phaB2-phaC2 del*	Subagyo et al. [37]
pk18msΔhad	pK18mobsacB derivative; *had del*	Segawa et al. [20]
pk18msΔpaaH1	pK18mobsacB derivative; *paaH1 del*	Segawa et al. [20]
pk18msΔcrt2	pK18mobsacB derivative; *crt2 del*	Segawa et al. [20]
pk18msΔA3330	pK18mobsacB derivative; *h16_A3330 del*	This study
pk18msΔbktB	pK18mobsacB derivative; *bktB del*	This study
pk18msΔphaJ4a	pK18mobsacB derivative; *phaJ4a del*	Kawashima et al. [25]
pK18ms∆fadB’	pK18mobsacB derivative; *fadB’ del*	Insomphun et al. [12]
pBPP-ccr_Me_-phaJ4a-emd	pBBR ori, *mob*,* P*_*phaP1*_, *ccr*_*Me*_, *phaJ4a*, *emd*_*Mm*_, *T*_*rrnB*_	Insomphun et al. [14]
pBPP-ccr_Me_-phaJ_Ac_-emd	pBBR ori, *mob*, *P*_*phaP1*_, *ccr*_*Me*_, *phaJ*_*Ac*_, *emd*_*Mm*_, *T*_*rrnB*_	Insomphun et al. [14]

The postfix *del* indicates constructs for targeted gene deletion. *Ac*, *Aeromonas caviae*; *Me*, *Methylorubrum extorquen*s; *Mm*, *Mus musculus*. *phaC*_NSDG_, a gene encoding N149S/D171G mutant of PHA synthase from *A. caviae*

### Construction of recombinant *R. eutropha* strains

The glucose-utilizable strain NSDG-GG having *phaC*_NSDG_ was used as a parent strain to construct various deletion mutants in this study. The gene deletion in *R. eutropha* chromosome was carried out through homologous recombination using pk18mobsacB-based suicide vectors, where the targeted genes were *phaB1* (*h16_A2171*), *phaB2-C2* (*h16_A2002-A2003*), *phaB3* (*h16_A2171*), *had* (*h16_A0602*), *paaH1* (*h16_A0282*), *crt2* (*h16_A3307*), *bktB* (*h16_A1445*), *phaJ4a* (*h16_A1070*), *h16_A3330*, and *fadB’* (*h16_A0461*) (Additional file 1: Table S2). The deletion vectors for *bktB* and *h16_A3330* were constructed by inserting the respective fragments connecting the upstream and downstream regions of the target gene by inverse PCR. The details of the construction are described in the supplementary text and the sequences of oligonucleotide primers used for PCR amplification are shown in Additional file 1: Table S3. Deletion of other genes were conducted using vectors that had been constructed previously [14, 20, 25, 36]. The previously constructed pBPP-ccr_Me_-phaJ4a-emd and pBPP-ccr_Me_-phaJ_Ac_-emd [15, 34] were used to overexpress the genes for P(3HB-*co*-3HHx) synthesis.

Transconjugation of the mobilizable plasmids to *R. eutropha* strains were performed using *E. coli* S17-1 as the donor strain, as previously described [37]. In the cases of chromosomal modification, transconjugants into which the pk18mobsacB-based suicide vector in interest was integrated into the chromosome (pop-in strains) were selected on a Simmons Citrate Agar (BD diagnostics, Franklin Lakes, NJ, USA) plate medium containing 250 mg/L kanamycin. The integrants were plated on an NR agar medium containing 10% (w/v) sucrose for the second recombination event (pop-out strains). Sucrose-resistant isolates were selected based on PCR analysis to confirm the deleted allele. The transconjugants of *R. eutropha* harboring the mobilizable expression vectors were selected on the Simmons Citrate Agar plate medium containing 250 mg/L kanamycin.

### Production and analyses of PHA

PHA production by *R. eutropha* strains was carried out at 30 °C in a 500 mL Sakaguchi flask with 100 mL of a nitrogen-limited mineral salts (MB) medium, which composed of 9 g of Na_2_HPO_4_·12H_2_O, 1.5 g of KH_2_PO_4_, 0.5 g of NH_4_Cl, 0.2 g of MgSO_4_·7H_2_O, and 1 mL of trace element solution in 1 L of deionized water [37]. A filtered-sterilized solution of glucose was added to the medium to a final concentration of 1% (w/v). In this study, aerobic condition was defined by a reciprocal shaking at 120 strokes/min; meanwhile low-aerated cultivation was conducted under a low-shaking speed of 60 strokes/min. Unless otherwise stated, the medium composition and fermentation condition were fixed throughout the study. After cultivation for 120 h, the cells were harvested, washed with cold deionized water, and then lyophilized. The content and composition of intracellular PHA were determined by gas chromatography after methanolysis of the dried cells in the presence of 15% (v/v) sulfuric acid, as previous described [38].

### Supplementary Information

**Additional file 1: Table S1.** Progress on P(3HB-*co*-3HHx) production from structurally unrelated carbon sources by recombinant bacteria. **Table S2.** Genes involved in this study. **Table S3.** Sequences of primers used in this study. **Table S4.** Effect of shaking condition on P(3HB-*co*-3HHx) biosynthesis by parent NSDG-GG and the *phaB*-deleted mutants. **Table S5.** Time profile of PHA accumulation in *R. eutropha* strains under aerobic and microaerobic cultivation. **Table S6.** Effects of nitrogen and oxygen limitation on PHA production by *R. eutropha* ΔB1 under microaerobic condition. **Table S7.** Effects of gene disruption of endogenous genes potentially related to 3HHx incorporation into PHAs in R. eutropha ΔB1-based strains under microaerobic condition. **Table S8.** Effects of introduction of Ccr-Emd along with PhaJ4a/PhaJAc on P(3HB-*co*-3HHx) biosynthesis by *R. eutropha* NSDG-GG and the *phaB*-deleted strains under aerobic and microaerobic conditions.

## Data Availability

All data generated and analyzed during this study were included in this manuscript.
