# Supplementary material for: Microaerobic insights into production of polyhydroxyalkanoates containing 3-hydroxyhexanoate via native reverse β-oxidation from glucose in Ralstonia eutropha H16
Source: Microb Cell Fact. 2024 Jan 14;23:21. doi: 10.1186/s12934-024-02294-4 (PMC10788006; doi:10.1186/s12934-024-02294-4)
Supplement: Supplementary file 1 — Additional file 1: Table S1. Progress on P(3HB-co-3HHx) production from structurally unrelated carbon sources by recombinant bacteria. Table S2. Genes involved in this study. Table S3. Sequences of primers used in this study. Table S4. Effect of shaking condition on P(3HB-co-3HHx) biosynthesis by parent NSDG-GG and the phaB-deleted mutants. Table S5. Time profile of PHA accumulation in R. eutropha strains under aerobic and microaerobic cultivation. Table S6. Effects of nitrogen and oxygen limitation on PHA production by R. eutropha ΔB1 under microaerobic condition. Table S7. Effects of gene disruption of endogenous genes potentially related to 3HHx incorporation into PHAs in R. eutropha ΔB1-based strains under microaerobic condition. Table S8. Effects of introduction of Ccr-Emd along with PhaJ4a/PhaJAc on P(3HB-co-3HHx) biosynthesis by R. eutropha NSDG-GG and the phaB-deleted strains under aerobic and microaerobic conditions. [file 12934_2024_2294_MOESM1_ESM.docx]

*Microbial Cell Factories*

Doi: 10.1186/s12934-024-02294-4

Supplementary material

**Microaerobic insights into production of polyhydroxyalkanoates containing 3-hydroxyhexanoate via native reverse *β*-oxidation from glucose in *Ralstonia eutropha* H16**

Kai-Hee Huong, Izumi Orita, Toshiaki Fukui^*^

School of Life Science and Technology, Tokyo Institute of Technology, 4259 Nagatsuta, Midori-ku, Yokohama 226-8501, Japan.

*Corresponding author:

Toshiaki Fukui

School of Life Science and Technology,

Tokyo Institute of Technology.

Tel/Fax: 045-924-5766

E-mail: [tfukui@bio.titech.ac.jp](mailto:tfukui@bio.titech.ac.jp)

| Host strain | 3HHx-CoA supply pathway | Relevant gene(s) | Carbon source | PHA  (wt%) | 3HHx (mol%) | Ref. |
| --- | --- | --- | --- | --- | --- | --- |
| *Pseudomonas putida* GPp104 | Hydrolysis of acyl-ACP to free fatty acids followed by β-oxidation | *phaB_Re_*, *phaG_Pp_* | Glucose | 12.5–18.6 | 4.6–6.8 | 1 |
| *Aeromonas hydrophila*  4AK4 | Hydrolysis of acyl-ACP to free fatty acids followed by β-oxidation | ‘*tesA* | Gluconate | 10.7–14.7 | 12.8–19.2 | 1 |
| *Escherichia coli*  LZ05, LZ06 | Reverse β-oxidation driven by Ter*_Td_* | *phaCJ_Ac_*, *phaAB_Re_*, *ter_Td_*, *fadBA_Ec_*  Deletion of thioesterase genes | Glucose | 11.9–12.9 | 12.8–13.2 | 2 |
| *Ralstonia eutropha*  PHB^-^4 | Reverse β-oxidation driven by Ccr*_Sc_* | *phaCJ_Ac_-ccr_Sc_* | Fructose | 39 | 1.6 | 3 |
| *R. eutropha* MF01ΔB1 | Reverse β-oxidation  driven by Ccr*_Sc_-Emd_Mm_* | *∆phaC::phaC*_NSDG_, *∆phaA::bktB*,  *∆phaB1*, *ccr_Me_*-*phaJ4a_Re_*-*emd_Mm_* | Fructose | 47.9 | 22.2 | 4 |
| *R. eutropha* MF01ΔB1 | Reverse β-oxidation  driven by Ccr*_Me_-Emd_Mm_* | *∆phaC::phaC*_NSDG_, *∆phaA::bktB*,  *∆phaB1*, *ccr_Me_-phaJ4a_Re_*-*emd_Mm_* | CO_2_ | 85.8 | 47.7 | 5 |
| *R. eutropha*  NSDG-GG-HC | Reverse β-oxidation  driven by Ccr*_Me_-Emd_Mm_* | *∆phaC::phaC*_NSDG_, ∆*phaB1*::*had*-*crt2*,  *ccr_Me_*-*phaJ4a_Re_/phaJ_Ac_*-*emd_Mm_*,  glucose utilization  glycerol utilization | Fructose/  Glucose  Glycerol | 68.7–78.1  36.8 | 12.1–26.0  3.1 | 6 |
| *R. eutropha* 144SR | Reverse β-oxidation  driven by Ccr*_Sc_-Emd_Mm_* | *∆phaC::phaC*_NSDG_, ∆*phaAB1*, *ccr_Me_*-*emd_Mm_*  Strong promoter for *phaJ4b* and *bktB*  glucose utilization, sucrose utilization | Sucrose | 56.8 | 27.2 | 7 |
| *R. eutropha* 145SR | Reverse β-oxidation  driven by Ccr*_Sc_-Emd_Mm_* | *∆phaC::phaC*_NSDG_*,* ∆*phaAB1*, *ccr_Me_-emd_Mm_*, *phaJ_Ac_*  Strong promoter for *phaJ4b* and *bktB*  glucose utilization | Glucose | 68.5 | 4.6 | 8 |
| *E. coli* JM109 | Reverse β-oxidation  driven by Ccr*_Me_-Emd_Mm_* | *phaC*_NSDG_*J_Ac_*, *phaAB1_Re_*, *ccr_Me_*-*phaJ4a_Re_*-*emd_Mm_*, *had_Re_*-*crt2_Re_*-*bktB* | Glucose | 41.0 | 14.0 | 9 |

**Supplementary Table S1:** Progress on P(3HB-*co*-3HHx) production from structurally unrelated carbon sources by recombinant bacteria

*ccr,* crotonyl-CoA carboxylase/reductase gene; *crt2*, (*S*)-enoyl-CoA hydratase (crotonase) gene; *emd,* ethylmalonyl-CoA decarboxylase gene; *fadA*, 3-ketoacyl-CoA thiolase gene; *fadB*, bifunctional (*S*)-enoyl-CoA hydratase/(*S*)-3-hydroxyacyl-CoA dehydrogenase gene; *had*, (*S*)-3HB-CoA dehydrogenase gene; *phaA*, *bktB*, β-ketothiolase genes; *phaB*, acetoacetyl-CoA reductase gene; *phaC*, PHA synthase gene, *phaC*_NSDG_, N149S/D171G double mutant of PHA synthase derived from *A. caviae*; *phaG*, 3-hydroxyacyl-ACP thioesterase gene; *phaJ*, (*R*)-enoyl-CoA hydratase gene; *ter,* *trans*-enoyl-CoA reductase gene; ‘*tesA,* truncated thioesterase gene. *Ac, Aeromonas caviae*; *Ah, Aeromonas hydrophila*; *Ec, Escherichia coli*; *Me, Methylorubrum extorquens*; *Mm, Mus musculus; Pp, Pseudomonas putida*; *Re, Ralstonia eutropha*; *Sc, Streptomyces cinnamonensis*; *Te, Treponema denticola*.

**References:**

1. Qiu Y-Z, Han J, Guo J-J, Chen G-Q. Production of poly(3-hydroxybutyrate-*co*-3-hydroxyhexanoate) from gluconate and glucose by recombinant *Aeromonas hydrophila* and *Pseudomonas putida*. *Biotechnol. Lett.* 2002, 27, 1381–1386.

2. Wang Q, Luan Y, Cheng X, Zhuang Q, Qi S. Engineering of *Escherichia coli* for the biosynthesis of poly(3-hydroxybutyrate-*co*-3-hydroxyhexanoate) from glucose. *Appl. Microbiol. Biotechnol.* 2015, 99, 2593–2602.

3. Fukui T, Abe H, Doi Y. Engineering of *Ralstonia eutropha* for production of poly(3-hydroxybutyrate-*co*-3-hydroxyhexanoate) from fructose and solid-state properties of the copolymer. Biomacromol. 2002*,* 3*,* 618–624.

4. Insomphun C, Xie H, Mifune J, Kawashima Y, Orita I, Nakamura S, Fukui T. Improved artificial pathway for biosynthesis of poly(3-hydroxybutyrate-*co*-3-hydroxyhexanoate) with high C_6_-monomer composition from fructose in *Ralstonia eutropha*. Metab. Eng. 2015*,* 27*,* 38–45.

5. Tanaka K, Yoshida K, Orita I, Fukui T. Biosynthesis of poly(3-hydroxybutyrate-*co*-3-hydroxyhexanoate) from CO_2_ by a recombinant *Cupriavidus necator*. Bioeng. 2021*,* 8*,* 179.

6. Zhang M, Kurita S, Orita I, Nakamura S, Fukui,T. Modification of acetoacetyl-CoA reduction step in *Ralstonia eutropha* for biosynthesis of poly(3-hydroxybutyrate-*co*-3-hydroxyhexanoate) from structurally unrelated compounds. Microb. Cell Fact. 2019*,* 18, 147*.*

7. Arikawa H, Matsumoto K, Fujiki T. Polyhydroxyalkanoate production from sucrose by *Cupriavidus necator* strains harboring csc genes from *Escherichia coli* W. Appl. Microbiol. Biotechnol. 2017, 101, 7497–7507.

8. Arikawa H, Sato S. Impact of various β-ketothiolase genes on PHBHHx production in *Cupriavidus necator* H16 derivatives. Appl. Microbiol. Biotechnol. 2002, 106, 3021–3032.

9. Saito S, Imai R, Miyahara Y, Nakagawa M, Orita I, Tsuge T, Fukui T. Biosynthesis of poly(3-hydroxybutyrate-*co*-3-hydroxyhexanoate) from glucose by *Escherichia coli* through butyryl-CoA formation driven by Ccr-Emd combination. Front. Bioeng. Biotechnol. 2022*,* 10, 888973*.*

**Supplementary Table S2:** Genes involved in this study

| Gene | Corresponding enzyme |
| --- | --- |
| *phaC*_NSDG_ | N149S/D171G double mutant of PHA synthase 1 derived from *Aeromonas caviae* |
| *phaC2* (*h16_A2003*) | PHA synthase 2 |
| *phaB1* (*h16_A1439*) | NADPH-acetoacetyl-CoA reductases 1, 2, and 3 |
| *phaB2* (*h16_A2002*) |  |
| *phaB3* (*h16_A2171*) |  |
| *had* (*h16_A0602*) | NAD^+^-(*S*)-3HB-CoA dehydrogenase (adh-short family) |
| *paaH1* (*h16_A0282*) | NAD^+^-(*S*)-3HB-CoA dehydrogenase (3HCDH family) |
| *crt2* (*h16_A3307)* | (*S*)-2-enoyl-CoA hydratase (crotonase) |
| *fadB’* (*h16_A0461*) | Bifunctional (*S*)-3HB-CoA dehydrogenase/(*S*)-enoyl-CoA hydratase |
| *bktB* (*h16_A1445*) | Broad substrate range-β-ketothiolase |
| *phaJ4a* (*h16_A1070*) | Medium chain-specific (*R*)-2-enoyl-CoA hydratase |
| *phaJ_Ac_* | Short chain-specific (*R*)-2-enoyl-CoA hydratase derived from *Aeromonas caviae* |
| *h16_A3330* | Putative acryloyl-CoA reductase |
| *ccr_Me_* | Crotonyl-CoA carboxylase/reductase derived from *Methylorubrum extorquens* |
| *emd_Mm_* | Ethylmalonyl-CoA decarboxylase derived from *Mus musculus* (codon-optimized) |

**Supplementary Table S3:** Sequences of primers used in this study

| Primer | Sequence (5’🡪3’) |
| --- | --- |
| pK18-bktB-Up820-5_A | GTCGACTCTAGAGGATCCCCTGGAACGGCCGCCCGCTCAA |
| pK18-bktB-Down820-3_F | CGAATTCGAGCTCGGTACCCCGAATCGGACACGGCGGTTT |
| dBktB-Inv-5 | GGTTAGCCTTGCGCCTTGCTTCGCT |
| dBktB-Inv-3 | GACTTTGTCTCCATGAGGTTATGT |
| A3330_flank_F | GCTCTAGACGGACTGCACCGGTTGTTCTCGTTGC |
| A3330_flank_R | GCTCTAGACAGCGGCGTGTAGGTTTCCTCGGC |
| A3330_Inv_F | GGTGTCTCCTTTGTCGCCTGCCGGCGAT |
| A3330_Inv_R | TCGCGCACCGTCGCCCTGATGCACC |
| A3330_conf_F | GCTGCCGGACAACCATGTGTCGATGGTCTGGG |
| A3330_conf_R | GCATCGGCCTTGACGCCGAACGTTTTGAACAGC |

Construction of plasmids.

pk18msΔbktB

A coding region of *bktB* (*h16_A1445*) along with the upstream and downstream flanking regions (approximately 820 bp each) was amplified from genomic DNA of *R. eutropha* H16 by PCR using a primer set of pK18-bktB-Up820-5_A/pK18-bktB-Down820-3_F that consisted of overlapping sequence of pK18mobsacB. The resulting fragment was seamlessly cloned into pK18mobsacB vector via *in vivo* iVEC3 cloning system (Nozaki and Niki J. Biotechnol. 201:e00660-18, 2019) using *E. coli* ME9806 strain (MG1655 Δ*hsdR* Δ*endA* Δ*recA*). The plasmid was extracted and subjected to inverse PCR using a primer set dBktB-Inv-5/dBktB-Inv-3 to remove the *bktB* coding region, and the resulting fragment of the upstream and downstream regions of *bktB* flanking to the plasmid backbone was 5’-phosphorylated and self-ligated.

pk18msΔA3330

A coding region of *h16_A3330* along with the upstream and downstream flanking regions (approximately 1,045 bp each) was amplified from genomic DNA of *R. eutropha* H16 by PCR using a primer set of A3330_flank_F/A3330_flank_R. The resulting fragment was ligated into pK18mobsacB vector and transformed into *E. coli* S17-1. The plasmid was extracted and subjected to inverse PCR using a primer set A3330_Inv_F/A3330_Inv_R to remove the *h16_A3330* coding region, and the resulting fragment of the upstream and downstream regions of *A3330* flanking to the plasmid backbone was 5’-phosphorylated and self-ligated.

**Supplementary Table S4:** Effect of shaking condition on P(3HB-*co*-3HHx) biosynthesis by parent NSDG-GG and the *phaB*-deleted mutants

| Strain | Dry cell  weight  (g/L) | Residual cell weight (g/L) | PHA  (wt%) | PHA  (g/L) | 3HHx  (mol%) |
| --- | --- | --- | --- | --- | --- |
| Aerobic |  |  |  |  |  |
| NSDG-GG | 4.47 ± 0.03 | 0.66 ± 0.05 | 85.3 ± 1.2 | 3.81 ± 0.08 | 0 |
| ΔB1 | 2.23 ± 0.31 | 0.81 ± 0.16 | 63.8 ± 4.6 | 1.42 ± 0.22 | 0.08 ± 0.02 |
| ΔB1ΔB3 | 1.10 ± 0.08 | 0.82 ± 0.05 | 25.8 ± 0.8 | 0.28 ± 0.97 | 0.85 ± 0.12 |
| ΔB1ΔB3ΔB2-C2 | 1.16 ± 0.03 | 0.85 ± 0.01 | 26.7 ± 0.5 | 0.31 ± 0.02 | 0.86 ± 0.07 |
| Microaerobic |  |  |  |  |  |
| NSDG-GG | 4.27 ± 0.03 | 0.60 ± 0.05 | 85.4 ± 1.0 | 3.65 ± 0.02 | 0.20 ± 0.01 |
| ΔB1 | 2.34 ± 0.08 | 0.79 ± 0.11 | 66.8 ± 4.6 | 1.58 ± 0.11 | 1.84 ± 0.09 |
| ΔB1ΔB3 | 2.01 ± 0.02 | 0.74 ± 0.07 | 63.0 ± 3.6 | 1.26 ± 0.07 | 2.85 ± 0.04 |
| ΔB1ΔB3ΔB2-C2 | 1.73 ± 0.17 | 0.81 ± 0.11 | 53.3 ± 3.7 | 0.92 ± 0.11 | 3.87 ± 0.35 |

Cultivation condition: 1% (w/v) glucose; 100 mL MB; 120 strokes/min (aerobic) or 60 strokes/min (microaerobic); 120 h; 30°C

**Supplementary Table S5:** Time profile of PHA accumulation in *R. eutropha* strains under aerobic and microaerobic cultivation

| Strain | PHA (g/L) | |
| --- | --- | --- |
|  | **Aerobic**  **(120 s/m)** | **Microaerobic**  **(60 s/m)** |
| NSDG-GG |  |  |
| 3 day (72 h) | 3.63 ± 0.25 | 2.27 ± 0.11 |
| 4 day (96 h) | 3.63 ± 0.05 | 3.23 ± 0.14 |
| 5 day (120 h) | 3.81 ± 0.08 | 3.64 ± 0.02 |
| ΔB1 |  |  |
| 3 day (72 h) | 1.27 ± 0.12 | 1.06 ± 0.04 |
| 4 day (96 h) | 1.27 ± 0.15 | 1.35 ± 0.14 |
| 5 day (120 h) | 1.55 ± 0.01 | 1.60 ± 0.09 |
| ΔB1ΔB3 |  |  |
| 3 day (72 h) | 0.19 ± 0.01 | 0.71 ± 0.07 |
| 4 day (96 h) | 0.25 ± 0.01 | 0.99 ± 0.06 |
| 5 day (120 h) | 0.26 ± 0.01 | 1.15 ± 0.16 |
| ΔB1ΔB3ΔB2-C2 |  |  |
| 3 day (72 h) | 0.21 ± 0.03 | 0.66 ± 0.02 |
| 4 day (96 h) | 0.37 ± 0.04 | 0.83 ± 0.01 |
| 5 day (120 h) | 0.36 ± 0.03 | 0.91 ± 0.11 |

Cultivation condition: 1% (w/v) glucose; 100 mL MB; 120 strokes/min (aerobic) or 60 strokes/min (microaerobic); 72 or 96 or 120 h; 30°C

**Supplementary Table S6:** Effects of nitrogen and oxygen limitation on PHA production by *R. eutropha* ΔB1 under microaerobic condition

| NH_4_Cl  (g/L) | Dry cell  weight  (g/L) | Residual  cell weight  (g/L) | PHA  (wt%) | PHA  (g/L) | 3HHx  (mol%) |
| --- | --- | --- | --- | --- | --- |
| Aerobic |  |  |  |  |  |
| 0.5 | 2.22 ± 0.43 | 0.86 ± 0.17 | 61.2 ± 0.2 | 1.36 ± 0.26 | 0.09 |
| 1.0 | 3.12 ± 0.01 | 1.84 ± 0.05 | 41.0 ± 1.4 | 1.28 ± 0.04 | 0.03 |
| 2.0 | 2.71 ± 0.01 | 2.38 ± 0.03 | 12.3 ± 0.8 | 0.33 ± 0.02 | 0 |
| Microaerobic |  |  |  |  |  |
| 0.5 | 2.37 ± 0.05 | 0.72 ± 0.02 | 69.7 ± 1.4 | 1.65 ± 0.07 | 1.86 ± 0.02 |
| 1.0 | 3.37 ± 0.04 | 1.71 ± 0.06 | 49.1 ± 2.2 | 1.65 ± 0.09 | 1.69 ± 0.02 |
| 2.0 | 3.65 ± 0.11 | 2.11 ± 0.04 | 42.2 ± 0.4 | 1.54 ± 0.06 | 1.72 ± 0.01 |

Cultivation condition: 1% (w/v) glucose; 100 mL MB; 120 strokes/min (aerobic) or 60 strokes/min (microaerobic); 120 h; 30°C with different concentration of NH_4_Cl (0.5, 1.0, 2.0 g/L)

**Supplementary Table S7:** Effects of gene disruption of endogenous genes potentially related to 3HHx incorporation into PHAs in *R. eutropha* ∆B1-based strains under microaerobic condition

| Strain | Dry cell  weight  (g/L) | Residual cell weight  (g/L) | PHA (wt%) | PHA  (g/L) | 3HHx  (mol%) |
| --- | --- | --- | --- | --- | --- |
| ΔB1 | 2.33 ± 0.09 | 0.76 ± 0.06 | 67.3 ± 1.6 | 1.56 ± 0.05 | 1.92 ± 0.21 |
| ΔB1Δ*bktB* | 2.59 ± 0.06 | 0.68 ± 0.10 | 73.8 ± 3.1 | 1.91 ± 0.04 | 0.60 ± 0.02 |
| ΔB1Δ*phaJ4a* | 2.13 ± 0.10 | 0.80 ± 0.01 | 62.2 ± 1.9 | 1.33 ± 0.10 | 0 |
| ΔB1Δ*had* | 2.46 ± 0.11 | 0.84 ± 0.03 | 65.8 ± 0.4 | 1.62 ± 0.08 | 1.55 ± 0.12 |
| ΔB1Δ*paaH1* | 2.56 ± 0.03 | 0.80 ± 0.03 | 68.6 ± 1.5 | 1.76 ± 0.06 | 0.59 ± 0.07 |
| ΔB1Δ*crt2* | 1.64 ± 0.07 | 0.75 ± 0.05 | 68.7 ± 2.0 | 1.64 ± 0.07 | 1.81 ± 0.03 |
| ΔB1Δ*paaH1*Δ*had* | 2.88 ± 0.13 | 0.82 ± 0.07 | 71.6 ± 1.3 | 2.06 ± 0.06 | 0.20 ± 0.05 |
| ΔB1Δ*paaH1*Δ*had*Δ*crt2* | 2.80 ± 0.01 | 0.83 ± 0.04 | 70.2 ± 1.6 | 1.96 ± 0.04 | 0.23 ± 0.03 |
| ΔB1Δ*fadB’* | 2.38 ± 0.08 | 0.84 ± 0.03 | 64.5 ± 0.8 | 1.53 ± 0.06 | 1.84 ± 0.07 |
| ΔB1Δ*A3330* | 2.40 ± 0.05 | 0.84 ± 0.04 | 64.8 ± 1.7 | 1.55 ± 0.06 | 1.52 ± 0.09 |

Cultivation condition: 1% (w/v) glucose; 100 mL MB; 60 strokes/min; 120 h; 30°C

**Supplementary Table S8:** Effects of introduction of Ccr-Emd along with PhaJ4a/PhaJ*_Ac_* on P(3HB-*co*-3HHx) biosynthesis by *R. eutropha* NSDG-GG and the *phaB*-deleted strains under aerobic and microaerobic conditions.

|  | Dry cell  weight (g/L) | Residual cell weight (g/L) | PHA  (wt%) | PHA  (g/L) | 3HHx  (mol%) |
| --- | --- | --- | --- | --- | --- |
| NSDG-GG |  |  |  |  |  |
| *Aerobic* |  |  |  |  |  |
| (no plasmid) | 4.47 ± 0.03 | 0.66 ± 0.05 | 85.3 ± 1.2 | 3.81 ± 0.08 | 0 |
| pBPP-Ccr-phaJ_4a_-Emd | 3.81 ± 0.10 | 0.71 ± 0.12 | 81.3 ± 3.5 | 3.10 ± 0.19 | 3.67 ± 1.14 |
| pBPP-Ccr-phaJ_Ac_-Emd | 3.81 ± 0.12 | 0.59 ± 0.15 | 84.5 ± 4.2 | 3.22 ± 0.24 | 3.40 ± 0.84 |
| *Microaerobic* |  |  |  |  |  |
| (no plasmid) | 4.27 ± 0.03 | 0.62 ± 0.05 | 85.4 ± 1.1 | 3.64 ± 0.02 | 0.20 ± 0.01 |
| pBPP-Ccr-phaJ_4a_-Emd | 3.69 ± 0.21 | 0.87 ± 0.12 | 76.4 ± 1.9 | 2.82 ± 0.09 | 6.36 ± 0.10 |
| pBPP-Ccr-phaJ_Ac_-Emd | 3.44 ± 0.08 | 0.89 ± 0.01 | 73.9 ± 0.8 | 2.54 ± 0.09 | 9.82 ± 0.73 |
| ΔB1 |  |  |  |  |  |
| *Aerobic* |  |  |  |  |  |
| (no plasmid) | 2.23 ± 0.31 | 0.81 ± 0.16 | 63.8 ± 4.6 | 1.42 ± 0.21 | 0.07 ± 0.02 |
| pBPP-Ccr-phaJ_4a_-Emd | 2.97 ± 0.04 | 0.93 ± 0.03 | 68.8 ± 0.6 | 2.05 ± 0.02 | 32.5 ± 0.4 |
| pBPP-Ccr-phaJ_Ac_-Emd | 3.05 ± 0.06 | 0.85 ± 0.02 | 72.2 ± 1.3 | 2.20 ± 0.08 | 11.1 ± 0.4 |
| *Microaerobic* |  |  |  |  |  |
| (no plasmid) | 2.33 ± 0.09 | 0.76 ± 0.06 | 67.3 ± 1.6 | 1.56 ± 0.05 | 1.92 ± 0.21 |
| pBPP-Ccr-phaJ_4a_-Emd | 3.33 ± 0.18 | 0.87 ± 0.06 | 73.8 ± 1.4 | 2.46 ± 0.14 | 32.2 ± 0.2 |
| pBPP-Ccr-phaJ_Ac_-Emd | 3.23 ± 0.17 | 0.97 ± 0.07 | 70.0 ± 1.8 | 2.26 ± 0.14 | 18.2 ± 1.1 |
| ΔB1 ΔB3 |  |  |  |  |  |
| *Aerobic* |  |  |  |  |  |
| (no plasmid) | 1.10 ± 0.08 | 0.82 ± 0.05 | 25.8 ± 0.8 | 0.28 ± 0.03 | 0.86 ± 0.12 |
| pBPP-Ccr-phaJ_4a_-Emd | 3.07 ± 0.02 | 0.88 ± 0.06 | 71.3 ± 1.7 | 2.19 ± 0.04 | 24.9 ± 0.3 |
| pBPP-Ccr-phaJ_Ac_-Emd | 3.20 ± 0.02 | 0.87 ± 0.07 | 72.8 ± 1.8 | 2.34 ± 0.04 | 16.0 ± 2.7 |
| *Microaerobic* |  |  |  |  |  |
| (no plasmid) | 2.00 ± 0.02 | 0.74 ± 0.07 | 63.0 ± 3.6 | 1.26 ± 0.07 | 2.85 ± 0.04 |
| pBPP-Ccr-phaJ_4a_-Emd | 2.91 ± 0.02 | 1.01 ± 0.01 | 65.2 ± 0.6 | 1.90 ± 0.03 | 35.4 ± 0.4 |
| pBPP-Ccr-phaJ_Ac_-Emd | 2.95 ± 0.11 | 0.96 ± 0.01 | 67.3 ± 1.5 | 1.99 ± 0.12 | 18.0 ± 1.1 |
| ΔB1 ΔB3 ΔB2-C2 |  |  |  |  |  |
| *Aerobic* |  |  |  |  |  |
| (no plasmid) | 1.16 ± 0.03 | 0.31 ± 0.13 | 26.7 ± 0.5 | 0.31 ± 0.01 | 0.86 ± 0.07 |
| pBPP-Ccr-phaJ_4a_-Emd | 2.97 ± 0.03 | 0.92 ± 0.07 | 68.9 ± 2.2 | 2.05 ± 0.05 | 30.8 ± 2.2 |
| pBPP-Ccr-phaJ_Ac_-Emd | 3.12 ± 0.01 | 0.87 ± 0.07 | 72.2 ± 2.4 | 2.25 ± 0.08 | 14.5 ± 0.5 |
| *Microaerobic* |  |  |  |  |  |
| (no plasmid) | 1.73 ± 0.17 | 0.81 ± 0.11 | 53.3 ± 3.7 | 0.92 ± 0.11 | 3.87 ± 0.35 |
| pBPP-Ccr-phaJ_4a_-Emd | 3.30 ± 0.22 | 0.91 ± 0.03 | 72.3 ± 1.8 | 2.39 ± 0.22 | 37.9 ± 2.0 |
| pBPP-Ccr-phaJ_Ac_-Emd | 3.08 ± 0.22 | 0.86 ± 0.02 | 72.1 ± 1.4 | 2.23 ± 0.20 | 18.1 ± 1.3 |

Cultivation condition: 1% (w/v) glucose; 100 mL MB; 120 strokes/min (aerobic) or 60 strokes/min (microaerobic); 120 h; 30°C
